# Erratum to “From goal to outcome: Analyzing the progression of biomedical sciences PhD careers in a longitudinal study using an expanded taxonomy”

**DOI:** 10.1096/fba.2023-00134

**Published:** 2023-12-01

Brown, A. M., Meyers, L. C., Varadarajan, J., Ward, N. J., Cartailler, J. P., Chalkley, R. G., Gould, K. L., and Petrie, K. A. From goal to outcome: Analyzing the progression of biomedical sciences PhD careers in a longitudinal study using an expanded taxonomy. *FASEB BioAdvances* 2023;5:427–452. https://doi.org/10.1096/fba.2023‐00072

This article is part of the Bioscience Careers special collection. It was added to the collection after publication.

