# Supplementary material for: From goal to outcome: Analyzing the progression of biomedical sciences PhD careers in a longitudinal study using an expanded taxonomy
Source: FASEB Bioadv. 2023 Oct 5;5(11):427–52. doi: 10.1096/fba.2023-00072 (PMC10626162; doi:10.1096/fba.2023-00072)
Supplement: Supplementary file 3 — Table S2: Career area at Y1, Y3, and Y5 compared to Y10 [file FBA2-5-427-s001.pdf]

S2 Table: Career area at Y1, Y3, and Y5 compared to Y10

		Career area at Y10						Total
		Academic Research	For-profit Research	Govt./Nonprofit Research	Teaching	AMO	Further Education	
Career area at Y1	Academic Research	172	61	33	43	108	1	418
	For-profit Research	1	15	1	1	8		26
	Govt./Nonprofit Research	29	16	10	3	26	1	85
	Teaching	2	-	-	20	3	-	25
	AMO	-	1	-	-	60	-	61
	Further Education	3	-	-	-	30	6	39
Career area at Y3	Academic Research	168	43	24	29	61	1	326
	For-profit Research	4	32	2	2	19	-	59
	Govt./Nonprofit Research	27	17	18	2	23	1	88
	Teaching	3	-	-	33	4	-	40
	AMO	2	1	-	-	104	-	107
	Further Education	3	-		1	24	6	34
Career area at Y5	Academic Research	175	29	10	16	32	-	262
	For-profit Research	3	46	3	-	21	-	73
	Govt./Nonprofit Research	19	15	29	1	12	1	77
	Teaching	4	-	-	49	2	-	55
	AMO	3	3	2	1	155	1	165
	Further Education	3	-	-	-	13	6	22
